# Roflumilast N-Oxide Prevents Cytokine Secretion Induced by Cigarette Smoke Combined with LPS through JAK/STAT and ERK1/2 Inhibition in Airway Epithelial Cells

**DOI:** 10.1371/journal.pone.0085243

**Published:** 2014-01-08

**Authors:** Tatiana Victoni, Florence Gleonnec, Manuella Lanzetti, Hermann Tenor, Samuel Valença, Luis Cristovão Porto, Vincent Lagente, Elisabeth Boichot

**Affiliations:** 1 UMR991 INSERM/Université de Rennes 1, Rennes, France; 2 Laboratório de Reparo Tecidual, DHE/IBRAG/UERJ, Rio de Janeiro, Brasil; 3 Nycomed GmbH, Konstanz, Germany; University of Giessen Lung Center, Germany

## Abstract

Cigarette smoke is a major cause of chronic obstructive pulmonary disease (COPD). Airway epithelial cells and macrophages are the first defense cells against cigarette smoke and these cells are an important source of pro-inflammatory cytokines. These cytokines play a role in progressive airflow limitation and chronic airways inflammation. Furthermore, the chronic colonization of airways by Gram-negative bacteria, contributes to the persistent airways inflammation and progression of COPD. The current study addressed the effects of cigarette smoke along with lipolysaccharide (LPS) in airway epithelial cells as a representative *in vitro* model of COPD exacerbations. Furthermore, we evaluated the effects of PDE4 inhibitor, the roflumilast N-oxide (RNO), in this experimental model. A549 cells were stimulated with cigarette smoke extract (CSE) alone (0.4% to 10%) or in combination with a low concentration of LPS (0.1 µg/ml) for 2 h or 24 h for measurement of chemokine protein and mRNAs and 5–120 min for protein phosphorylation. Cells were also pre-incubated with MAP kinases inhibitors and Prostaglandin E_2_ alone or combined with RNO, before the addition of CSE+LPS. Production of cytokines was determined by ELISA and protein phosphorylation by western blotting and phospho-kinase array. CSE did not induce production of IL-8/CXCL8 and Gro-α/CXCL1 from A549 cells, but increase production of CCL2/MCP-1. However the combination of LPS 0.1 µg/ml with CSE 2% or 4% induced an important production of these chemokines, that appears to be dependent of ERK1/2 and JAK/STAT pathways but did not require JNK and p38 pathways. Moreover, RNO associated with PGE_2_ reduced CSE+LPS-induced cytokine release, which can happen by occur through of ERK1/2 and JAK/STAT pathways. We report here an in vitro model that can reflect what happen in airway epithelial cells in COPD exacerbation. We also showed a new pathway where CSE+LPS can induce cytokine release from A549 cells, which is reduced by RNO.

## Introduction

Chronic obstructive pulmonary disease (COPD) is a major and a growing cause of morbidity and mortality worldwide. COPD is characterized by airflow limitation that is not fully reversible [1]. The airflow limitation is usually progressive and associated with an abnormal inflammatory response of the lungs [2]. The major triggering factor is cigarette smoking, which accounts for 80–90% of the COPD cases. The cigarette smoke causes airway inflammation by activating epithelial cells and macrophages, which by releasing proteases and free oxygen radicals cause injury of parenchyma tissue. These cells can also release inflammatory mediators, including cytokines and chemokines such as IL-8/CXCL8, monocyte chemotactic peptide-1 (MCP-1/CCL2) and Growth-related oncoprotein-α (Gro-α/CXCL1). These chemokines play a role in mechanisms leading to inflammatory process in airways and progressive airflow limitation [3], [4], [5]. Besides, there is now increasing amount of evidence that chronic colonization of airways by respiratory pathogens, predominantly gram-negative bacteria, contributes to the progression of COPD and is also responsible for the persistent airway inflammation [6], [7].

Several signaling pathways, such as mitogen activated protein kinase (MAPK) control the expression of these chemokines as demonstrated by taking advantage of selective inhibitors or siRNA strategies [8], [9], [10], [11]. Indeed, inhibitors of ERK 1/2 and p38 MAP kinases the decreased the release of cytokines induced by cigarette smoke in airway epithelial cells [12], [13]. Other protein kinases may be involved in inflammatory responses like Scr family kinases, JAKs (Janus kinases) as well as their downstream transcription factors of the STAT (signal transducer and activator of transcription) family [14], [15].

Phosphodiesterase 4 (PDE4) inhibitors, in view of their antiinflammatory effects, have recently been confirmed as a potential novel therapeutic approach for the treatment of exacerbations in COPD [16]. PDE4 inhibitors that prevent the degradation of cAMP will enhance the anti-inflammatory action of this second messenger. Neutrophils, macrophages, CD4+ and CD8+ T-lymphocytes, epithelial cells or fibroblasts in the lung and airways express PDE4 and their functions are favorably modulated by PDE4 inhibitors such as roflumilast [17], which may finally translate into a clinical benefit in COPD. Indeed, roflumilast robustly reduced the rate of acute exacerbations in the frequent exacerbation phenotype of COPD as documented in large clinical trials [16].

*In vivo*, roflumilast mitigates lung infiltration by inflammatory cells (neutrophils, macrophages, dendritic cells, B and T-cells) and airspace enlargement in mice exposed to tobacco smoke over a period of 6 months [18]. *In vitro*, the PDE4 inhibitor enhances ciliary beat frequency and preserves the ciliated cell phenotype compromised by tobacco smoke extract in differentiated human bronchial epithelial cells [19] and reduces MUC5AC expression [12].

The airway epithelium is the first line of defense against inhaled environmental particles such as tobacco smoke and bacteria. Thus, airway epithelial cells represent an important source of cytokines and chemokines in the context of COPD [20], [21]. The precise role of epithelial cells in the complex interplay between tobacco smoke, pathogens and the inflammatory microenvironment has not yet been fully explored. The aim of this study was to evaluate the effects of cigarette smoke extracts associate with LPS, a constituent of gram negative bacteria cell walls on airway epithelial cells. Furthermore, the effects of the PDE4 inhibitor roflumilast N-oxide (RNO) the active metabolite of roflumilast, in chemokines release and kinases activation were explored in this experimental model.

To this end, we have examined the effect of combination of cigarette smoke extract (CSE) with a low concentration of lipolysaccharide (LPS) on human alveolar epithelial type II like cells (A549). We showed that CSE with LPS further enhanced the release and expression of several chemokines by A549 cells, namely IL-8/CXCL8, MCP-1/CCL2 and Gro-α/CXCL1 to which an activation of the ERK1/2 and JAK/STAT pathways may have involved. Moreover, RNO associated with Prostaglandin E_2_ (PGE_2_) reduced the release of these chemokines that can be occur through inhibition of ERK1/2 and JAK/STAT pathways.

## Materials and Methods

### Reagents

Roflumilast N-oxide (RNO) was provided by Nycomed (Konstanz, Germany). F-12K Nutrient Mixture Kaighn's Modification cell culture medium, antibiotics, glutamine and trypsin-EDTA were purchased from Invitrogen (Eugene, OR, USA). Fetal calf serum (FCS) was from Hyclone (Logan, UT, USA). Lipolysaccharide from E. coli 055:B5, Thiazolyl Blue Tetrazolium Blue (MTT), SB-203580, SP-600125 and U0126 were purchased from Sigma-Aldrich (St. Louis, MO, USA). The specific antibodies against phospho-(p44/42) ERK1/2, (p44/42) ERK1/2, phospho-p38 MAP kinase, p38 MAP kinase, phospho-SAPK/JNK, SAPK/JNK, were purchased from Cell Signaling Technology (Beverly, MA, USA). Acrylamide, SDS, Tris, HEPES and BSA were purchased from Eurobio (Les Ulis, France). Bradford protein assay and precision plus protein dual color standards were purchased from Bio-Rad (Hercules, CA, USA).

### Cell culture

The human alveolar epithelial cell line (A549) was purchased from American Type Culture Collection (Manassas, VA, USA) and cultured in F-12K supplemented with 10% FCS, 1% antibiotics, 2 mM L-glutamine and 10 mM HEPES at 37°C and at 5% CO2. 1×10^5^ cells were transferred to 24-well plates and grown to confluence for the experiments.

### Cell culture and treatments

CSE was prepared as previously reported [22], [23]. In brief two 2R1 cigarettes (U Kentucky, USA) was bubbled through 20 ml F-12K Nutrient Mixture Kaighn's Modification and the resulting suspension (100% CSE) was filtered through a 0.2 µm filter and diluted with complete media. A549 cells were stimulated with CSE (0.4% to 10%) associate or not with LPS (0.1 µg/ml) for 2 h or 24 h. Cells were also pre-incubated with PGE_2_ (10 nM) alone or associate with RNO (1 nM or 1 µM) or vehicle for 2 h before the addition of CSE+LPS. To explore possible signaling pathways involved A549 cell were pre-incubated with either the p38 kinase inhibitor SB203580 (10 µM and 20 µM) or the JNK inhibitor SP600125 (2 µM and 4 µM) or the MEK inhibitor U0126 (3 µM and 5 µM) for 2 h before being stimulated with CSE+LPS. All experiments were performed in serum-free medium, triplicate and repeated at least 3 times. At the end of the incubation period culture supernatants were harvested and stored at -80°C until further analysis.

### Measurement of chemokine protein and mRNAs

The concentrations of IL-8/CXCL8, MCP-1/CCL2, Gro-α/CXCL1 in the culture supernatants were measured by ELISA from R&D Systems (Abingdon, United Kingdom). Total RNAs were isolated from A549 cells using a commercially available kit containing DNase (Promega, Madison, WI, USA). RNA quantity and purity were assessed with a Nanodrop ND-1000 spectrophotometer (Nyxor Biotech, Paris, France). Total RNAs (1 µg) was reverse-transcribed into cDNA using the High-Capacity cDNA Archive kit (Applied Biosystems, Foster City, CA). Real-time quantitative RT-PCR was performed by the fluorescent dye SYBR Green methodology using the SYBR Green PCR Master Mix (Applied Biosystems) and the StepOnePlus™ real-time PCR system (Applied Biosystems). Primer pairs for each transcript were chosen with NCBI software http://www.ncbi.nlm.nih.gov. IL-8/CXCL8 primers forward 5′-AAG AAA CCA CCG GAA GGA AC-3′, reverse 5′-AAA TTT GGG GTG GAA AGG TT-3′; MCP-1/CCL2 primers forward 5′-TGT CCC AAA GAA GCT GTG ATC-3′, reverse 5′-ATT CTT GGG TTG TGG AGT GAG-3′; Gro-α/CXCL1 primers forward 5′-AAC CGA AGT CAT AGC CAC AC-3′, reverse 5′CCT CCC TTC TGG TCA GTT G3′; GAPDH primers forward 5′-GGC ATG GAC TGT GGT CAT GAG-3′, reverse 5′-TGC ACC ACC AAC TGC TTA GC-3′). Amplification curves were read with the StepOne software V2.1 using the comparative cycle threshold method. The relative quantification of the steady-state mRNA levels was normalized against GAPDH mRNA.

### Evaluation of protein kinase phosphorylation by Western blotting

A549 cells were incubated with medium alone, CSE 4%, LPS 0.1 µg/ml or the combination of both for 5, 15, 30, 60 and 120 min. Then, cells were washed with PBS and lysed with lysis buffer (Novagen, San Diego, CA, USA) containing 1% protease inhibitor cocktail and phosphatase inhibitor cocktail (Roche, Mannheim, Germany) for 15 min on ice. Equal amounts of cell lysate (50 µg) were separated by a 4–10% SDS-PAGE gel and then transferred into a nitrocellulose membrane, which was further incubated for 1 hour with 5% BSA or 5% w/v nonfat dry milk in TBS containing 0.1% Tween 20 and then for 2 h at room temperature with an antibody specific. After washing, the membranes were incubated for 2 h with a horseradish peroxidase conjugated anti-mouse/rabbit antibody. Blots were then incubated with an enhanced chemiluminescence solution for 1 min and exposed.

### Human Phosphoprotein Array

Cells were pre-incubated with RNO (1 nM) and PGE_2_ (10 nM) or vehicle for 2 h before the addition of CSE (4%), LPS (0.1 µg/ml) and CSE+LPS. Cell lysates (500 µg of total proteins per array) were applied to the phosphoprotein array following the manufacturer's instructions (Proteome Profiler Human Phosphokinase Array kit, R&D Systems, Abingdon, United Kingdom).

### Viability assay by the tetrazolium salt method

Cytotoxicity of CSE tested on A549 cells was assessed by using the tetrazolium salt method (MTT) viability test. MTT was added to the culture medium at a final concentration of 0.5 mg/ml and incubated at 37°C for 2 h. The reaction product of MTT was extracted in dimethylsulfoxide (DMSO) and the OD was spectrophotometrically measured at 570 nm, with DMSO as a blank. Viability was expressed as percentage of the values (corresponding to 100%) of untreated cells.

### Statistical analysis

The results are expressed as means ± SEM. Analysis of treatment effects between groups was performed with a one-way ANOVA. Comparison of treatment interactions was done by tukey post test. For each analysis, *P* values less than 5% were considered statistically significant.

## Results

### Effects of the combination of LPS with CSE on the production of cytokines from epithelial cells

**Figure 1:**
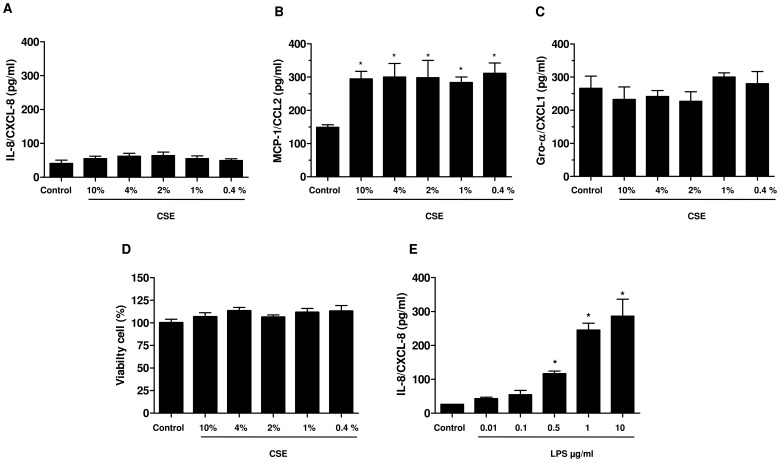
Effects of CSE (0.1%–10%) and LPS (10 ng/ml–10 µg/ml) on chemokine release and cell viability. Serum-starved A549 cells were incubated with medium alone (control) or with different concentration of CSE (0.1%–10%) and LPS (10 ng/ml–10 µg/ml) for 24 h. The culture supernatants were collected and the concentrations of IL-8/CXCL8, Gro-α/CXCL1 and MCP-1/CCL2 were measured by ELISA (A, B, C and D) and viability was determined by MTT test (E). The data represent the mean ± SEM of 3 experiments. * p<0.05 compared to control.

**Figure 2:**
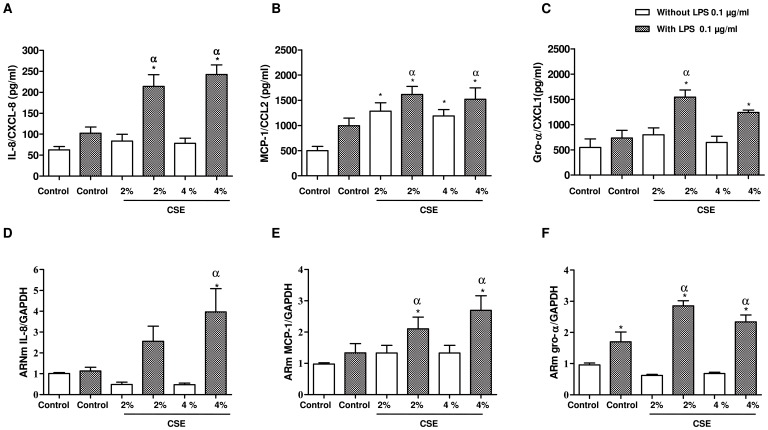
CSE associated with LPS induced chemokine release from A549 cells and mRNA expression. Serum-starved A549 cells were incubated with medium alone (control), CSE 2% or 4% (blank bars), LPS 0.1 µg/ml alone or in combination with 2 or 4% of CSE (hatched bars) for 24 h. The culture supernatants were collected and the concentrations of chemokines were measured by ELISA (A, B and C). The data represent the mean ± SEM of 7 experiments. A549 cells were stimulated for 2 h. mRNA expression was then determined by real-time quantitative RT-PCR (D, E and F). The results are normalized to the gene expression of GAPDH. The data represent the mean ± SEM of 4 experiments. * p<0.05 compared to control; α p<0.05 compared to LPS 0.1 µg/ml.

Incubation of A549 cells with different concentrations of CSE (0.1%–10%) for 24 h did not enhance the release of IL-8/CXCL8 and Gro-α/CXCL1 (Figure 1A and C), but significantly increase the release of MCP-1/CCL2 (Figure 1B). CSE whatever the concentrations did not induce toxicity (Figure 1D). As expected, LPS (10 ng/ml–10 µg/ml) increased the release of IL-8/CXCL8 from A549 cells in a dose dependant manner (Figure S1). For evaluating the effects of CSE associated with LPS in further experiments, the concentration of 0.1 µg/ml LPS was selected to not significantly enhance the release of IL-8/CXCL8 over control (Figure 1D). The incubation of A549 epithelial cells with CSE at 2% or at 4% in combination with LPS at 0.1 µg/ml for 24 h significantly enhanced the release of IL-8/CXCL8, MCP-1/CCL2 and Gro-α/CXCL1 into the culture medium that was more than additive for IL-8/CXCL8 and Gro-α/CXCL1 at both CSE concentrations. For instance, the basal release of IL-8/CXCL8 is 62.5±8.2 pg/ml accumulated in the culture medium after 24 h. In presence of CSE at 2% or LPS 0.1 µg/ml the release of IL-8/CXCL8 increased to 83.9±15.0 pg/ml or 102.5±14.92 pg/ml. However, in the presence of the combination of CSE at 2% with LPS at 0.1 µg/ml the secretion of IL-8/CXCL8 into the culture medium increased up to 214.1±27.7 pg/ml (Figure 2A). Furthermore, we also observed an increased of mRNA expression of IL-8/CXCL8, MCP-1/CCL2 and Gro-α/CXCL1 when epithelial cells were stimulated with CSE 2% or 4% in combination with LPS after 2 h of incubation (Figure 2D-F). Though LPS at 0.1 µg/ml is able to increased mRNA expression of Gro-α/CXCL1, the increased of expression of this chemokine induced by LPS+CSE appears significantly more important (Figure 2F).

### The release of chemokines induced by the combination of CSE and LPS was contingent on ERK1/2 but did not require JNK and p38 pathways

**Figure 3:**
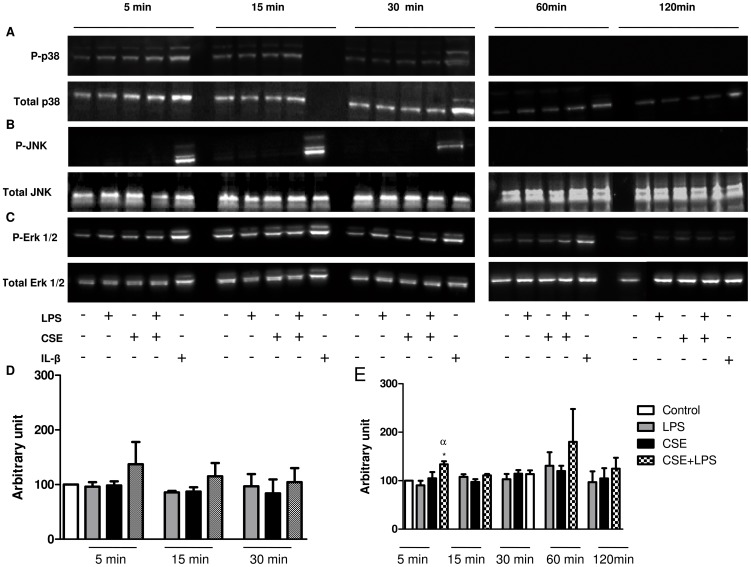
Effects of CSE and LPS alone or in combination in ERK1/2, p38 and JNK activation. Serum-starved A549 cells were incubated with serum free medium alone (control, Ctrl), LPS 0.1 µg/ml, CSE 4% or CSE 4% associated with LPS 0.1 µg/ml for 5, 15, 30, 60 or 120 min. Total cell lysates were immunoblotted with antibodies specific for phospho-p38 kinase and total p38 kinase (*A*), phospho-JNK and total JNK (*B*) or phospho ERK1/2 and total ERK1/2 (*C*). A549 cells stimulated with IL-1β (7 ng/ml) were used as positive controls. The data represent the mean ± SEM of 3 experiments. * p<0.05 compared to control; α p<0.05 compared to LPS 0.1 µg/ml.

**Figure 4:**
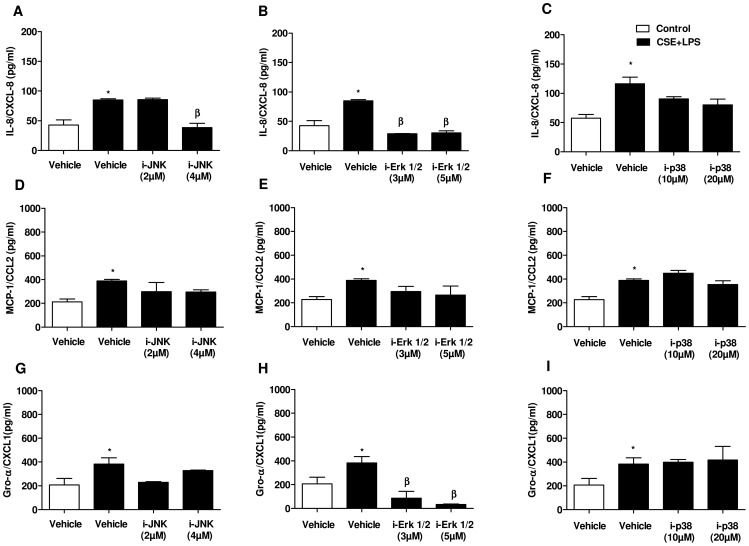
Effect of ERK 1/2, p38 and JNK inhibitor on chemokine release induce by CSE+LPS. Serum-starved A549 cells were preincubated with serum-free medium alone (control: blank bars), vehicle (0.1% DMSO), the p38 MAPKinhibitor SB203580 (10 µM and 20 µM), the MEK1/2 inhibitor U0126 (3 µM and 5 µM) or the JNK inhibitor SP600125 (2 µM and 4 µM) for 2 h and then stimulated with CSE (4%) together with LPS (0.1 µg/ml) for 24 h (black bars). At the end of the incubation period culture supernatants were collected for IL-8/CXCL8, MCP-1/CCL2 and Gro-α/CXCL1 quantification by ELISA. Data are expressed as means ± SEM of 3 independent experiments. * p<0.05 compared to control; β p<0.05 compared to vehicle.

To analyse the signalling pathways likely involved in CSE+LPS-induced chemokine release, the expression of phosphorylated forms of p38 kinase, JNK and ERK 1/2 were analysed by western blotting. Stimulation of A549 cells with CSE (4%) or LPS (0.1 µg/ml) alone or in combination over 5 to 120 min neither increased p38 nor JNK phosphorylation (Figure 3A and B). The incubation of epithelial cells with CSE and LPS alone are not able to increases significantly the expression of the active form of ERK 1/2 (Figure 3 E). On the other hand, the combination CSE+LPS results in a rapid and transient increase of ERK 1/2 activation observed at 5 min (Figure 3C and E). To confirm the involvement of MAP kinases in CSE+LPS induced release of chemokines, we investigated whether specific inhibitors of ERK1/2, p38 and JNK could reduce the release of various chemokines induced by CSE+LPS. Consistent with the results of western blotting, the MEK1/2 inhibitor U0126 (3 or 5 µM) abolished the CSE+LPS-induced release of IL-8/CXCL8 (Figure 4B) and Gro-α/CXCL1 (Figure 4H) below baseline (p<0.05) while the release of MCP-1/CCL2 (Figure 4E) was not significantly reduced. The p38 MAPK inhibitor SB203580 at 10 µM or 20 µM did not affect CSE+LPS-induced IL-8/CXCL8, MCP-1/CCL2 and Gro-α/CXCL1 release (Figure 4C, 4F and 4I). The JNK inhibitor, SP600125, at 4 µM but not at 2 µM significantly reduced the CSE+LPS-induced release of IL-8/CXCL8 (Figure 4A), but it has no effect in MCP-1/CCL2 and Gro-α/CXCL1 release (Figure 4D and 4G).

### Activation of the JAK/STAT pathway following stimulation of A549 epithelial cells with CSE and LPS

**Figure 5:**
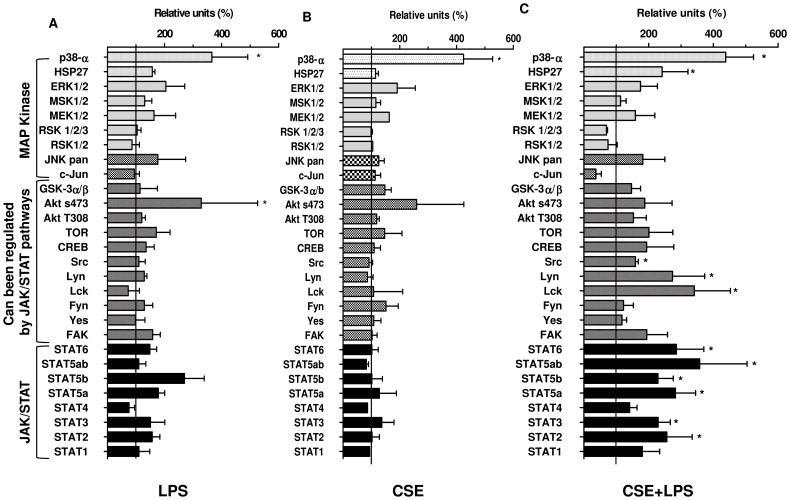
Effects of CSE and LPS alone or in combination on selected phosphoproteins in A549 cells. Cells were incubated for 40-free medium alone (control), LPS 0.1 µg/ml (A), CSE 4% (B), or CSE 4% and LPS 0.1 µg/ml (C). Then cell extracts were probed on human phosphoprotein arrays. Results representing the signal intensity (AU) were expressed as % of the unstimulated control. Data are expressed as means ± SEM of 3 independent experiments. * p<0.05 compared to control.

Due to the low degree of ERK activation and the lack effect of ERK 1/2 inhibitor in MCP-1/CCL2 release, we analyzed other intracellular signalling pathways following stimulation of A549 cells with CSE (4%) (Figure 5A), LPS (0.1 µg/ml) (Figure 5B) or their combination (Figure 5C). We used a commercially available human phosphoprotein array kit that allowed to semi-quantitatively assess the levels of phosphorylated representatives of the MAP Kinases, Src family and JAK/STAT pathways amongst others. Stimulation of epithelial cells with CSE+LPS (Figure 5C) for 40 min is able to activate eleven different kinases of 28 kinases studied. In contrast, CSE 4% (Figure 5A) and LPS 0.1 µg/ml (Figure 5B) alone affected only in 2 kinases of the 28 different kinases. Between the eleven activated kinases by CSE+LPS, we were observed that six of them (STAT2, STAT3, STAT5a, STAT5b, STATab, and STAT6) belong to JAK/STAT pathways. Moreover, other three kinases (Src, Lyn and Lck) which were increased by CSE+LPS can be involved in JAK/SAT pathways, also we observed no difference in ERK1/2 activation after LPS, CSE and CSE+LPS stimulation by 40 minutes. CSE, LPS and CSE+LPS are able to activate p38-α kinase, but no difference between groups was observed, supporting the data observed by western blotting.

**Figure 6:**
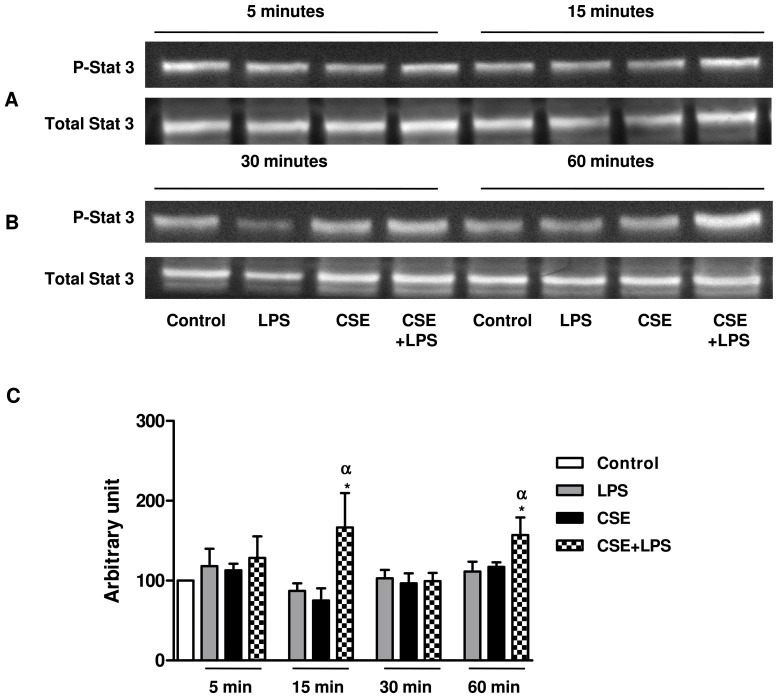
Effects of CSE and LPS alone or in combination on STAT3 phosphorylation in A549 cells. Serum-starved A549 cells were stimulated with serum free medium alone (control), LPS 0.1 µg/ml, CSE 4% or CSE 4% and LPS 0.1 µg/ml for 5, 15, 30 or 60 min. Total cell lysates were immunoblotted with antibodies specific for phospho STAT3 and total STAT3. Data are expressed as means ± SEM of 3 independent experiments.* p<0.05 compared to control; α p<0.05 compared to LPS 0.1 µg/ml.

In many cases, an activation of STAT kinase is not dependent of JAK, such as STAT3 that can be activated by other pathways, including Src kinases and EGF receptors[24]. Then, we investigated if the activation of STAT3 occurs at other time points by western blotting. An increase in phospho STAT3 activation was observed after 15 min and 60 min of incubation with CSE+LPS compared to the control, but this difference is not observed neither CSE nor with LPS alone (Figure 6).

### Effects of RNO on chemokine release and protein phosphorylation induced by the combination of CSE and LPS in A549 cells

**Figure 7:**
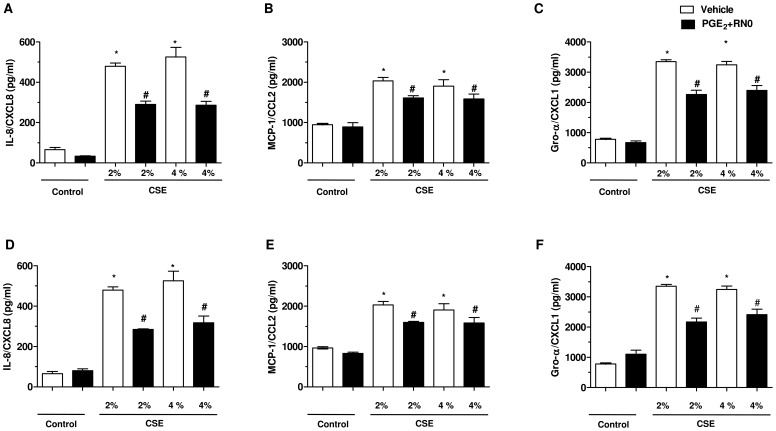
Roflumilast N-oxide associated with PGE_2_ partly inhibits chemokines release from A549 cells stimulated with CSE+LPS. Cells were preincubated with vehicle (blank bars) or 10 nM PGE_2_ with roflumilast N-oxide at 1 nM (A, B and C) or 1 µM (D, E and F) (hatched bars) for 2 h and then stimulated or not with CSE at 2% or 4% in combination with LPS at 0.1 µg/ml. After 24 hours cell culture supernatants were collected and chemokines were quantified by ELISA. Results are expressed as means ± SEM of 3 independent experiments. * p<0.05 compared to control (0.01% DMSO); # p<0.05 compared to vehicle.

**Figure 8:**
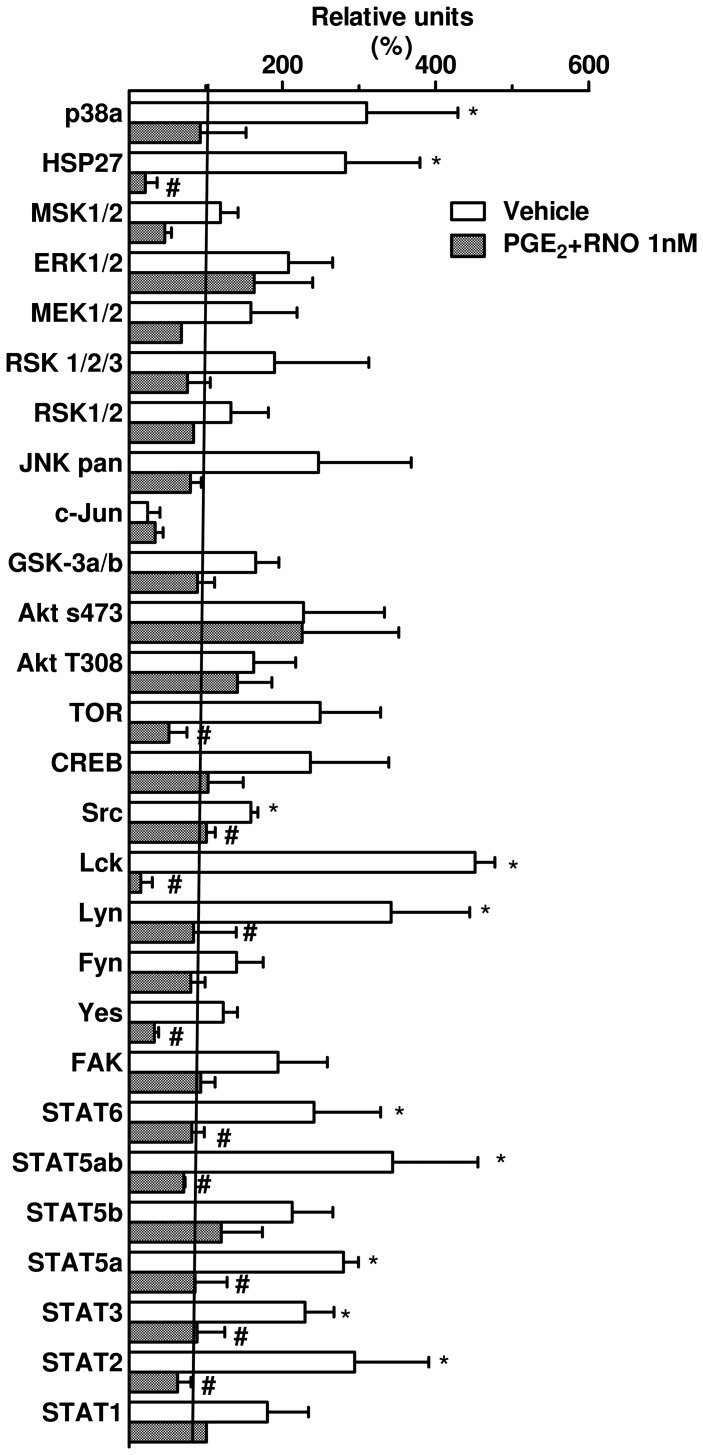
Effects of roflumilast N-oxide on selected phosphoproteins in A549 cells. Cells were preincubated for 2(blank bars) or PGE_2_ (10 nM) and roflumilast N-oxide (1 nM) (hatched bars) then co-stimulated with CSE (4%) and LPS (0.1 µg/ml). Cell extracts were probed on human phosphoprotein arrays. Results representing the signal intensity (AU) were expressed as % of the unstimulated control. Data are expressed as means ± SEM of 3 independent experiments. * p<0.05 compared to control; # p<0.05 compared to respective vehicle.

Finally, we investigated the effects of a PDE4 inhibitor, RNO in conjunction with PGE_2_ (10 nM) on the release of chemokines and the phosphorylation of a range of proteins following incubation of A549 cells with the combination of CSE (at 2% or 4%) and LPS (0.1 µg/ml). The treatment of epithelial cells with RNO at 1 nM (Figure 7A, B and C) or 1 µM (Figure 7 D, E and F) combined with PGE_2_ (10 nM) elicited a partial, but significant inhibition of the release of IL-8/CXCL8, MCP-1/CCL2 and Gro-α/CXCL1 into the culture medium of A549 cells stimulated with CSE+LPS. In contrast PGE_2_ alone does not modify the cytokine release (Figure S2). Whether RNO (1 nM) along with PGE_2_ (10 nM) would affect the JAK/STAT pathways was next investigated. RNO markedly reduced the signal intensity for seven of the eleven kinases increased by CSE+LPS. In the fact, RNO is able to decrease the activation of STAT2, STAT3, STAT5a, STAT5ab and STAT6 that were increased after exposure to CSE+LPS. Moreover, RNO decreased the signal intensity of MAPK and other kinases protein that can be regulated by STAT as Lck, Lyn, Yes, components of Scr-family, that were increased after exposure of A549 cells to CSE+LPS (Figure 8).

## Discussion

We have investigated the effects of combining CSE with a low concentration of LPS on human alveolar type II like epithelial cells, A549. We have observed that such a combination enhanced the mRNA expression and the release of several chemokines from A549 cells, in particular IL-8/CXCL8, MCP-1/CCL2 and Gro-α/CXCL1 by activation of the ERK1/2 and JAK/STAT pathways. This increase appears significantly higher when compared to CSE or LPS alone. We also observed that roflumilast N-oxide, a PDE4 inhibitor along with PGE_2_ partly reduced the release of chemokines induced by CSE+LPS involved the inhibition of ERK1/2 and JAK/STAT pathways.

Cigarette smoke impairs host immune defense and contributed to recurrent infections of the airways, that plays a crucial role in the progression of COPD [25]. In this study, we developed an *in vitro* model which can reflect what happen in COPD exacerbations where airway epithelial cells could face both tobacco smoke and bacterial pathogens, an amplification of the inflammatory response. We demonstrated that cigarette smoke alone is not sufficient to induce expression and the release of chemokines such as IL-8/CXCL8 and Gro-α/CXCL1. These results are in line with the study of Moodie (2004) and colleagues, showing that cigarette smoke-conditioned medium did not affect IL-8/CXCL8 or IL-6 expression in human lung epithelial cells *in vitro* [26]. On the other hand, the co-treatment of A549 cells by CSE in combination with a low concentration of LPS (0.1 µg/ml), which by its own did not affect the release of the chemokines, elicited a significant increase in IL-8/CXCL8, MCP-1/CCL2 and Gro-α/CXCL1 release after 24 h. The release of these chemiokines was associated with an increase of intracellular IL-8/CXCL8, MCP-1/CCL2 and Gro-α/CXCL1 mRNA expression. Our findings are consistent with those of Pace *et al*., (2008) who found an increase release of IL-8/CXCL8 from 16-HBE cells when LPS was associated with CSE [27]. However, Pace & colleagues used airway epithelial cells of bronchial origin while the A549 cells used in our study are of alveolar origin. In contrast, other studies report an inhibition of an LPS-induced cytokine release by CSE in airway epithelial cells [28], [29]. The concentrations of LPS used in the latter studies where 10-100-fold higher compared to the 0.1 µg/ml of LPS used in the current investigation that may result in a differential reponse of intracellular signaling pathways in addition to induction of cellular stress at high LPS concentration. Indeed, in healthy volunteers the inhalation of LPS at 5–50 µg/ml elicits neutrophilic airway inflammation [30]. Based on these studies, we conclude that concentrations of LPS between 1 µg/ml and 10 µg/ml would be extremely high for *in vitro* experiments with human cells. Moreover, LPS up to 1 µg/ml was not different from CSE 4% associated with LPS 1 µg/ml in chemokine release (data not shown).

The production of cytokines and chemokines may be regulated by a range of signal transduction pathways such as MAP Kinases [9] [31]. Thus, we examined by western blotting the phosphorylation pattern of p38 kinase, JNK and ERK1/2 in response to CSE+LPS. Although the generation of IL-8/CXCL8, MCP-1/CCL2 and Gro-α/CXCL1 was reported to be dependent on p38 kinase and JNK in several experimental models this was not the case under our conditions [9], [31], [32]. In our experimental model by using either western blotting or selective inhibitors, we found that the formation of IL-8/CXCL8, MCP-1/CCL2 and Gro-α/CXCL1 by A549 cells stimulated with CSE+LPS is independent from both JNK and p38 kinase activation. However, we observed a decrease in CSE+LPS-induced IL-8/CXCL8 release after incubation with the JNK inhibitor, SP600125 at 4 µM. It is therefore possible that at this concentration of SP600125 (4 µM), it might also exerts an inhibitory effect on the ERK1/2 pathway. Indeed, U0126, an inhibitor of the ERK1/2 pathway at concentrations of 3 and 5 µM decreased the release of IL-8/CXCL8 and Gro-α/CXCL1 from CSE+LPS-stimulated A549 cells indicating that the ERK1/2 pathway is involved in the generation of these chemokines under our conditions. On the other hand U0126 failed to reduce the release of MCP-1/CCL2. This observation and the relatively low degree of ERK1/2 activation suggest that other pathways may also contribute to the generation of these chemokines. Our hypothesis was confirmed by the analysis of phosphoprotein arrays following stimulation of A549 cells with CSE+LPS. For example the phosphorylation of six STAT proteins (STAT2, STAT3, STAT5a, STAT5b, STAT5ab and STAT6) was increased after exposure of A549 cells to CSE+LPS. These results suggest that in addition to ERK1/2, the JAK/STAT pathway is involved in the CSE+LPS-induced chemokine release. Indeed, the non-receptor tyrosine kinases such as JAK were implicated in the generation of cytokines or chimiokines [33], [34], also STATs can be activated independently of JAK, including Src kinases [24]. An example of STAT activation independently of JAK is the activation of STAT3 by epidermal growth factor receptor (EGFR) most notably by c-Src [24], [35]. EGFR transactivation pathway is involved in expression of proinflammatory cytokines, including IL-8/CXCL8 in response to cigarette smoke [36]. Moreover EGFR signaling is commonly involved in both the activation of extracellular signal-related kinases 1/2 (ERK1/2) and Scr-family, two other pathways that may be involved in CSE+LPS exposure [24], [37]. Based on these information, we focused our analysis on STAT3 and we observed an increase of expression of STAT3 at 15 and 60 min, demonstrating that the activation of STAT3 can occur at two different times. We suggest that the activation of Src family Kinases (Src, Lyn and Lck,) at 40 min of exposure with the combination of CSE+LPS as showed in protein arrays, might account for a second phosphorylation of STAT3 at 60 min as observed by western blotting. Moreover, Src have been shown to activate MAP Kinases in response to various stimuli [38], [39]. Similarly, Goyal *et al.*, (2010) showed that in a human intestinal epithelial cell line stimulated with fragments of *E. coli* the expression of IL-8/CXCL8 was operated through STAT3 and ERK1/2 activation [40]. We have hypothesized that activation of ERK1/2 pathway by the combination of CSE with LPS, may involved Src family and STAT3. This hypothesis is supported by Ovrerik et al. (46) suggesting that the activation of ERK1/2 is dependent of the Scr family for the IL-8/CXCL8 production by A549 stimulated with silica. In this condition, the low level of activation observed for ERK1/2 in our study, could be due to the delayed (more than 120 min) activation which is also dependent on the activation of Scr family and STAT3 pathways. But more experiments with different time point of ERK1/2 and STAT3 should be conducted to confirm this hypothesis.

The anti-inflammatory profile of PDE4 inhibitors is broadly documented [41] and it is support by the recent approval of one of them, roflumilast, in severe COPD where it reduces the risk of exacerbations [16]. Indeed, clinical trials reported a reduced exacerbation rate and an improved lung function with roflumilast in severe COPD [42]. The preclinical pharmacology of roflumilast and its active metabolite, roflumilast N-oxide with a wealth of anti-inflammatory effects has been described in numerous studies [43], [44]. Because an abnormal inflammatory response largely governs the pathophysiology of COPD and roflumilast is approved for patients with symptomatic, severe COPD with frequent exacerbations, then we explored the effects of roflumilast N-oxide in our *in vitro* experimental model of airway epithelial cells stimulated with CSE and LPS. We have shown that roflumilast N-oxide associated with PGE_2_ inhibits the release of chemokines after stimulation of A549 by CSE+LPS cells. The use of PGE_2_ seems necessary, indeed using in vitro models. Sabatini et al., (2010) have shown that PDE4 inhibition was dependent on the presence of PGE_2_ [45]. We assume that the epithelial cells do not produce large amounts of cAMP. The use of PGE_2_ allows the activation of adenylate cyclase and consequently an increase in intracellular cAMP, resulting in amplification of effect ant inflammatory of roflumilast in this model. Our results seem consistent with other authors that showed the inhibitory effects of roflumilast and roflumilast N-oxide on the release of cytokines and chemokines in vitro and in vivo[44]. Recently, roflumilast and roflumilast N-oxide were described to reduce the LPS-induced release of CCL2, 3, 4, CXCL10 and TNF-α from human lung macrophages [43]. However, the signaling pathways that might be affected by these molecules have been rarely studied although some authors have shown an inhibition of p38 and p44/42-MAPK by roflumilast [12]. Then, we investigated the inhibitory effect of roflumilast N-oxide on the phosphorylation of kinases. Roflumilast N-oxide at 1 nM (in the presence of 10 nM PGE_2_) prevented the phosphorylation of six STAT proteins and three kinases belonging at Scr family proteins. Based on our findings, we hypothesized that the inhibitory effect of roflumilast may have been caused by an inhibition of the phosphorylation of critical kinases mainly those of the JAK/STAT activation pathway. These data may suggest that others mechanisms are involved in anti-inflammatory activity of roflumilast.

## Conclusions

In conclusion, our study demonstrated that CSE+LPS can stimulate A549 epithelial cells to release cytokines which participate in the airway inflammatory process in COPD. Moreover, the combination of CSE and low concentration of LPS, but not LPS or CSE individually, is responsible for the activation JAK/STAT and ERK 1/2 pathways. We report a new pathway through which cigarette smoke associate with LPS can induced cytokines release on A549 cells. This in vitro model can reflect what happen in airway epithelial cells in COPD exacerbation context. We also showed that roflumilast N-oxide prevents cytokine secretion induced by CSE combined with LPS perhaps by JAK/STAT and ERK1/2 inhibition.

## Supporting Information

Figure S1 **Effects of LPS (10 ng/ml–10 µg/ml) on chemokine release from A549 cells.** Serum-starved A549 cells were incubated with medium alone (control) or with different concentration of LPS (10 ng/ml–10 µg/ml) for 24 h. The culture supernatants were collected and the concentrations of IL-8/CXCL8, Gro-α/CXCL1 and MCP-1/CCL2 were measured by ELISA. The data represent the mean ± SEM of 3 experiments. * p<0.05 compared to control.
(TIF)

Figure S2 **Effects of roflumilast N-oxide associated with PGE_2_ but not of PGE_2_ alone on chemokines release from A549 cells stimulated with CSE+LPS.** Cells were preincubated with vehicle, PGE_2_ alone or PGE_2_ associated with roflumilast N-oxide at 1 nM for 2 h and then stimulated or not with CSE at 2% or 4% in combination with LPS at 0.1 µg/ml. After 24 hours cell culture supernatants were collected and chemokines were quantified by ELISA. Results are expressed as means ± SEM of 3 independent experiments. * p<0.05 compared to control; # p<0.05 compared to vehicle.
(TIF)
