# Supplementary figures and images for: Roflumilast N-Oxide Prevents Cytokine Secretion Induced by Cigarette Smoke Combined with LPS through JAK/STAT and ERK1/2 Inhibition in Airway Epithelial Cells
Source: PLoS One. 2014 Jan 8;9(1):e85243. doi: 10.1371/journal.pone.0085243 (PMC3885699; doi:10.1371/journal.pone.0085243)

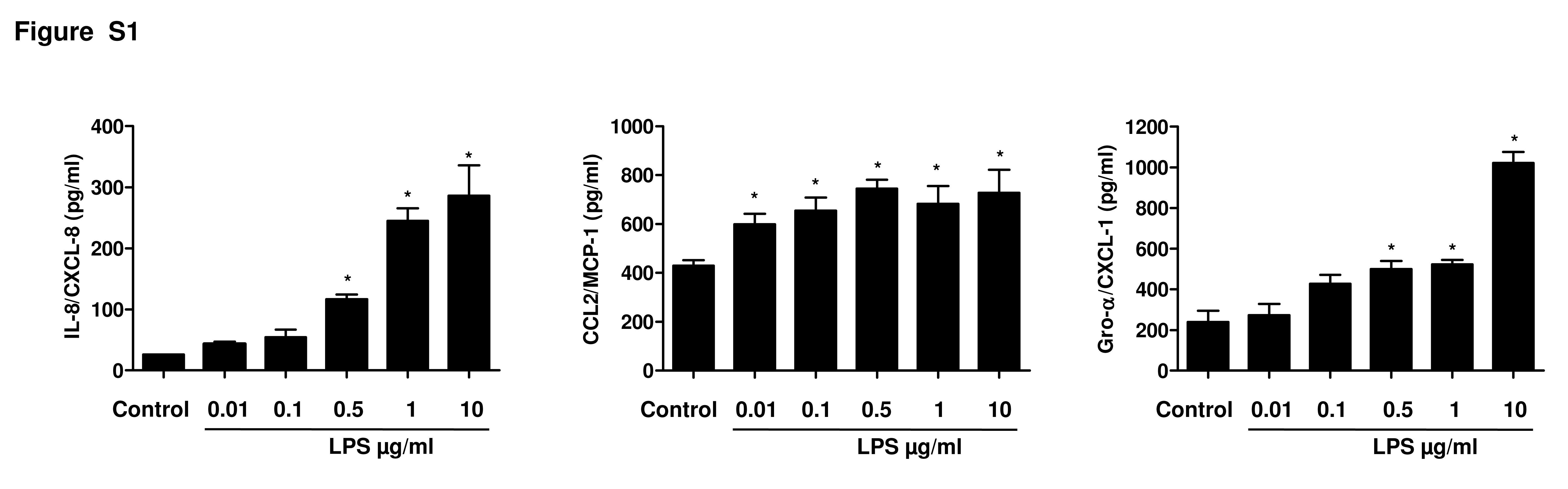

Supplement: Figure S1 — Effects of LPS (10 ng/ml–10 µg/ml) on chemokine release from A549 cells. Serum-starved A549 cells were incubated with medium alone (control) or with different concentration of LPS (10 ng/ml–10 µg/ml) for 24 h. The culture supernatants were collected and the concentrations of IL-8/CXCL8, Gro-α/CXCL1 and MCP-1/CCL2 were measured by ELISA. The data represent the mean ± SEM of 3 experiments. * p<0.05 compared to control. (TIF) [file pone.0085243.s001.tif]

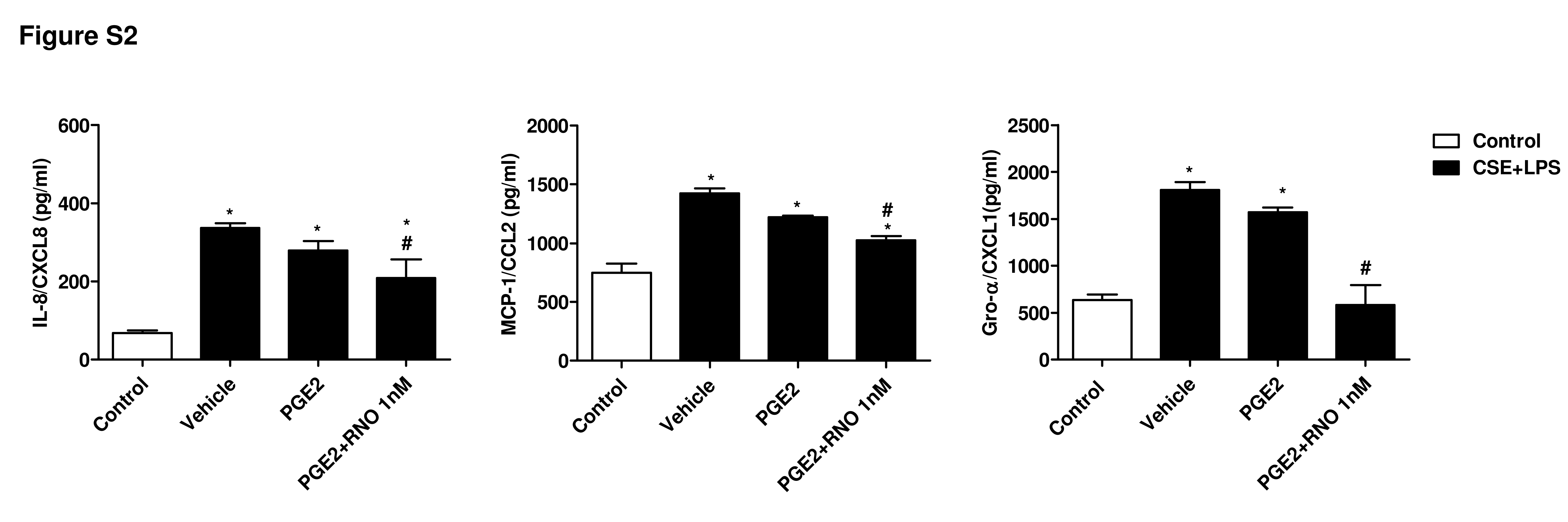

Supplement: Figure S2 — Effects of roflumilast N-oxide associated with PGE2 but not of PGE2 alone on chemokines release from A549 cells stimulated with CSE+LPS. Cells were preincubated with vehicle, PGE2 alone or PGE2 associated with roflumilast N-oxide at 1 nM for 2 h and then stimulated or not with CSE at 2% or 4% in combination with LPS at 0.1 µg/ml. After 24 hours cell culture supernatants were collected and chemokines were quantified by ELISA. Results are expressed as means ± SEM of 3 independent experiments. * p<0.05 compared to control; # p<0.05 compared to vehicle. (TIF) [file pone.0085243.s002.tif]
